# Public-health impact of outdoor air pollution for 2^nd^ air pollution management policy in Seoul metropolitan area, Korea

**DOI:** 10.1186/s40557-015-0058-z

**Published:** 2015-02-27

**Authors:** Jong Han Leem, Soon Tae Kim, Hwan Cheol Kim

**Affiliations:** Department of Occupational and Environmental Medicine, Inha University Hospital, 27 Inhang road Jung-gu, Incheon, 400-711 South Korea; Division of Environmental Engineering, Ajou University Woncheon-dong, Yeongtong-gu, Suwon, 443-749 South Korea

**Keywords:** Public health assessment, Air pollution, PM_2.5_, PM_10_, Mortality

## Abstract

**Objectives:**

Air pollution contributes to mortality and morbidity. We estimated the impact of outdoor air pollution on public health in Seoul metropolitan area, Korea. Attributable cases of morbidity and mortality were estimated.

**Methods:**

Epidemiology-based exposure-response functions for a 10 μg/m3 increase in particulate matter (PM_2.5_ and PM_10_) were used to quantify the effects of air pollution. Cases attributable to air pollution were estimated for mortality (adults ≥ 30 years), respiratory and cardiovascular hospital admissions (all ages), chronic bronchitis (all ages), and acute bronchitis episodes (≤18 years). Environmental exposure (PM_2.5_ and PM_10_) was modeled for each 3 km × 3 km.

**Results:**

In 2010, air pollution caused 15.9% of total mortality or approximately 15,346 attributable cases per year. Particulate air pollution also accounted for: 12,511 hospitalized cases of respiratory disease; 20,490 new cases of chronic bronchitis (adults); 278,346 episodes of acute bronchitis (children). After performing the 2^nd^ Seoul metropolitan air pollution management plan, the reducible death number associated with air pollution is 14,915 cases per year in 2024. We can reduce 57.9% of death associated with air pollution.

**Conclusion:**

This assessment estimates the public-health impacts of current patterns of air pollution. Although individual health risks of air pollution are relatively small, the public-health consequences are remarkable. Particulate air pollution remains a key target for public-health action in the Seoul metropolitan area. Our results, which have also been used for economic valuation, should guide decisions on the assessment of environmental health-policy options.

## Introduction

Urbanization and industrialization are ongoing worldwide. Air pollution accompanied by urbanization and industrialization has already become a major risk factor threatening human health. Fine PM (PM_2.5_) air pollution and mortality were linked in the Six Cities Study, which reported an association between PM_2.5_ and all cause, cardiopulmonary, and lung cancer mortality [1,2].

Research conducted during the past 20 years in the US, EU, and Asian countries has confirmed that outdoor air pollution contributes to morbidity and mortality [3-5]. Some effects may be related to short-term exposure [6,7], others have to be considered as contributions of long-term exposure. Although the mechanisms have not been fully explained, epidemiological evidence suggests that outdoor air pollution is a contributing cause of morbidity and mortality [8].

The recent Global Burden of Disease report estimated that 89% of the world’s population lived in areas with PM_2.5_ ambient levels above the World Health Organization (WHO) Air Quality Guideline of 10 μg/m3, and 32% lived in areas above the WHO Level 1 Interim Target of 35 μg/m3. East Asia was singled out, with an estimated 76% population exposure above the Level 1 Interim Target [9].

In 1998, the average PM_10_ concentration in Seoul was 78 μg/m3. According to the first metropolitan air pollution management policy, the average PM_10_ concentration in Seoul was 41 μg/m3 in 2012 [10]. Even with a dramatic decrease of PM_10_, the current level of PM_10_ still exerts a significant burden of disease on people in Korea.

The Korean government established the 2^nd^ metropolitan air pollution management plan during 2015–2024. In this plan, the goal of air quality is that PM_10_ reach 30 μg/m3 and PM_2.5_ reach 20 μg/m3.

Assessing public health benefit can be possible by risk assessment of air pollution. Up to now, public health assessment are not performed in Korea for monitoring air quality. In this study, we will assess public health benefit by calculating mortality and morbidity cases attributed to air pollution in the Seoul metropolitan area.

## Methods

### Design and participants

The impact assessment relies on calculating the attributable number of cases [11,12]. Cases of morbidity or mortality attributable to air pollution were derived for the health outcomes listed in Table 1. Outcomes were ignored if quantitative data were not available, if costing was impossible (e.g., valuing decrement in pulmonary function), and to prevent overlapping health measures from causing multiple counting of the same costs (e.g., emergency visits were not considered because they were partly included in the hospital admissions). We selected only PM_2.5_ or PM_10_ in order to derive the attributable cases because PM_2.5_ and PM_10_ are useful indicators of several sources of outdoor air pollution such as fossil-fuel combustion. Three data components are required for estimation of the number of cases attributed to outdoor air pollution in a given population: the exposure-response function; the frequency of the health outcome (e.g., the incidence or prevalence) and the level of exposure. The association between outdoor air pollution and health-outcome frequency is usually described with an exposure-response function (or effect estimate) that expresses the relative increase in adverse health for a given increment in air pollution.

**Table 1:** **Health outcome definition and source of data**

**Health Outcome**	**Definition**	**Source of exposure-response function**	**Source of population frequency**
Long-term mortality Adult age ≥ 30	Death rate, excluding violent death or accidents, >30 years	Dockery DW et al. [1]	National death certificate statistics for 2010 and 2024(prediction) (adults _30 years)
		Pope CA et al. [2]	
		Pope CA et al. [15]	
Respiratory hospital admissions (All age)	ICD9 460–519	Pope CA et al. [16]	National population statistics for 2010 and 2024(prediction)
	ICD9 466, 480–487, 493, 490–	Spix C et al. [17]	
	492, 494–496	Wordley J et al. [18]	
	ICD9 480–487, 490–496	Prescott GJ et al. [19]	
Cadiovascular hospital admissions (All age)	ICD9 410–436	Wordley J et al. [18]	National population statistics for 2010 and 2024(prediction)
	ICD9 390–459	Poloniecki JD et al. [20]	
	ICD9 390–459	Medina S et al. [21]	
	ICD9 410–414, 426–429, 434–440	Prescott GJ et al. [19]	
Lung cancer incidence	ICD9 162	Raaschou-Nielsen O et al. [26]	
Asthma attack (children < 15 years)	Lower respiratory symptoms, age 6–12 years	Roemer W et al. [27]	National population statistics for 2010 and 2024(prediction)
	Asthma, age 7–15 years	Segala C et al. [28]	
	Lower respiratory symptoms, age 7–13 years	Gielen MH et al. [29]	
Asthma attack (adults ≥ 15 years)	Wheeze, age 18–80 years	Dusseldorp A et al. [30]	National population statistics for 2010 and 2024(prediction)
	Shortness of breath, age 18–55 years	Hiltermann TJN et al. [31]	
	Wheeze, age 16–70 years	Neukirch F et al. [32]	
Chronic-bronchitis	Symptoms of cough and/or sputum production on most days, for at least 3 months per year, and for 2 years or more	Abbey D et al. [22]	National population statistics for 2010 and 2024(prediction)
Acute bronchitis (age ≤ 18)	Bronchitis in past 12 months, ages less than 18 years.	Dockery DW et al. [23]	National population statistics for 2010 and 2024(prediction)

### Baseline population, mortality and morbidity data

Population data for the Seoul metropolitan area from Statistics Korea is classified according to address and registration in the following age groups: 0–4, 5–9, 10–14, 15–19, 20–24, 25–29, 30–34, 35–39, 40–44, 45–49, 50–54, 55–59, 60–64, and 65+ years. Citizens’ residences in the Seoul metropolitan area were divided into 80 sections according to neighborhoods, forming basic administrative units such as Gu and Gun, used in city planning and management. Eighty sections were formed in order to identify site-specific exposure to air pollution and identify the areas with the greatest risk. 2024 population data were obtained from population prediction data of Statistics Korea. The health outcome definition and source of data are listed in Table 1. The total regional baseline mortality was retrieved from statistics on Korea (International Classification of Diseases–ICD-10, A00-Y98). ICD-9 code in previous studies were translated into ICD10. The morbidity calculations were performed using hospitalization data from the Korean Health Insurance, which covers the entire population and is the sole purchaser of health care services in the country. Hospitalizations due to two main disease groups were included in the calculations: cardiovascular (I00-I99) and respiratory causes (J00-J99). Cardiac admissions (I20-I25) and cerebrovascular admissions (I60-I69) were also used for the exposure-response work on cardiovascular hospitalizations.

### Exposure assessment

CMAQ (Comprehensive Multiscale Air Quality) [13] version 4.7.1 was used to simulate air quality over the Seoul Metropolitan Area (SMA) for a one-month period of each season in 2010and 2024; January, April, July, and October. The Nesting-down domains were composed of 27-km, 9-km, and 3-km horizontally resolved domains. The coarse domain (174x128 arrays) includes northeastern Asia; Korea, Japan, and most of China. The 9-km domain (67x82 arrays) includes all of South Korea and most of North Korea, and the finest domain (58x61 arrays) was set up to focus on the SMA. The SAPRC-99 (Statewide Air Pollution Research Center 99) [14] chemical mechanism for gas-phase chemistry and AERO5 for aerosol module were selected to represent model species over the region.

For meteorological simulation, WRF (Weather Research and Forecast) version 3.4.1 was utilized with NCEP (National Centers for Environmental Prediction) Final (FNL) Operational Global Analysis Model fields for initial and boundary conditions [13]. The WRF was configured to have 35 sigma layers up to 50 hpa, and the lowest layer thickness is around 30 m (sigma = 0.996). The WRF physical options are as follows: WRF Single-Moment 6-class scheme, Rapid Radiative Transfer Model longwave scheme, Goddard shortwave scheme, M-O surface layer scheme, Grell Cumulus scheme, and YSU PBL scheme. Meteorology-Chemistry Interface Program (MCIP) version 3.6 was then used for preparation of CMAQ-ready meteorological inputs. One-way nesting was applied during WRF and CMAQ simulations.

The CAPSS 2010 (Clean Air Protection Supporting System, 2010 base year), anthropogenic emissions inventory, was processed using SMOKE (Sparse Matrix Operation Kernel Emission) version 3.1, and biogenic emissions such as isoprene and terpenes were estimated using MEGAN (Model of Emissions of Gases and Aerosols from Nature) [15] version 2.04. The MICS-Asia emissions inventory was supplementary used for foreign emissions.

### Exposure-response functions, calculation of mortality and morbidity

To describe the long-term effect of air pollution on mortality, the broadly employed US ACS study relative risk RR = 1.06 (95% CI 1.02–1.11) per 10 μg/m3 increase of PM_2.5_ was used as the exposure-response relationship [16].

RR = 1.013 (95% CI 1.001–1.025) per 10 μg/m3 increase of PM_10_ was used for calculations of respiratory hospitalizations due to air pollution [17-20]. For cardiovascular hospitalizations we used a weighted average RR = 1.013 (95% CI 1.007–1.019) per 10 μg/m3 increase of PM_10_ based on the effect on cardiac and cerebrovascular admissions from a COMEAP meta-analysis [21,22]. For chronic bronchitis incidence, we used RR = 1.098 (95% CI 1.009–1.194) per 10 μg/m^3^ increase of PM10 based on one study, which reported the effect of PM on the incidence of chronic bronchitis among a population with very low rates of smoking [23].

For bronchitis episodes, we used RR = 1.306 (95% CI 1.135–1.502) per 10 μg/m^3^ increase of PM_10_ validated at several studies [24-26]. The meta-analyses showed a statistically significant association between risk of lung cancer and PM_10_ (hazard ratio [HR] 1 · 22 [95% CI 1 · 03-1 · 45] per 10 μg/m^3^ [27]. For asthma attacks that occurred in children younger than 15 years old, we used RR = 1.044 (95% CI 1.027–1.062) per 10 μg/m3 increase of PM_10_ [28-30]. For asthma attacks that occurred in adults older than 15 years old, we used RR = 1.039 (95% CI 1.019–1.059) per 10 μg/m3 increase of PM_10_ [31-33]. The cases (mortality and morbidity) were calculated in absolute and relative numbers for all sections in the Seoul Metropolitan area. The following equation was used:

$$ \varDelta \mathrm{Y}=\Big[Y0\left(\mathrm{e}{\hbox{-}}^{\upbeta \kern0.15em \cdot \kern0.15em \varDelta \mathrm{PM}2:5}\cdot \hbox{--} 1\right)\cdot POP $$

where *Y*0 is the baseline rate; *pop* the number of exposed persons; · the exposure-response function and △PM_2.5_ the estimated excess exposure.

For each outcome we selected studies from the peer-reviewed literature in order to derive the exposure-response function and the 95% CI. For inclusion, an adequate study design and published PM_10_ levels were required. Cross-sectional or cohort studies relying on two or three levels of exposure were omitted, as were ecological studies, given their inherent limitations. The health-outcome frequencies (mortality, prevalence, incidence, or person-days) may differ across countries; thus, national mortality and morbidity data were used (Table 2). For some morbidity data, epidemiological studies were the only source (bronchitis incidence from the Adventist Health and Smog Study [34], which was also used by Ostro and colleagues [35]. Annual mean outdoor PM_10_ had to be determined on a continuous scale. Although there is no evidence for any threshold, there are also no studies available where participants were exposed to PM_10_ below 20 μg/m3 (annual mean). This reference level also includes the natural background PM_10_. Thus, the health impact of air-pollution exposure below 20 μg/m3 was ignored. To derive the population exposure distribution, mean annual concentrations of PM_10_ were modelled for each area at a spatial resolution of 3 km × 3 km.

**Table 2:** **Effect estimate of health outcome and health outcome frequencies**

**Health Outcome**	**Effect estimate relative risk (95% CI)per 10 μg/m** ^**3**^ **PM**_**10**_	**Health outcome frequency per million inhabitants per year**
Long-term mortality Adult age ≥ 30	1 · 06 (1 · 02–1 · 11)*	5,950
Respiratory hospital admissions (All age)	1 · 013 (1 · 001–1 · 025)	15,630
Cadiovascular hospital admissions (All age)	1 · 013 (1 · 007–1 · 019)	15,430
Lung cancer incidence	1 · 22 (1 · 03-1 · 45)	287
Asthma attack (children < 15 years)	1 · 044 (1 · 027–1 · 062)	19,930
Asthma attack (adults ≥ 15 years)	1 · 039 (1 · 019–1 · 059)	24,570
Chronic-bronchitis (adults 25 years)	1 · 098 (1 · 009–1 · 194)	4,990
Acute bronchitis (age ≤ 18)	1 · 306 (1 · 135–1 · 502)	100,720

*per 10 μg/m^3^ PM_2.5_.

Using the exposure-response functions, expressed as relative risk (RR) per 10 μg/m^3^, and the health frequency per 1000 000 inhabitants, for each health outcome we calculated the attributable number of cases (D10) for an increase of 10 μg/m3 PM_10_, as: D10 = (RR-1)*P0 where P0 is the health frequency, given an baseline exposure E0 and RR is the mean exposure-response function across the studies used (Table 1). The exposure-response functions are usually log-linear. For small risks and across limited ranges of exposure log-linear and linear functions would provide similar results. However, if one may apply the method to populations with very large exposure ranges, the impact may be seriously overestimated on the log-linear scale. Thus, we derived the attributable number of cases on an additive scale. The study protocol was approved by the Institutional Review Boards of the Inha University College of Medicine.

## Results

**Figure 1:**
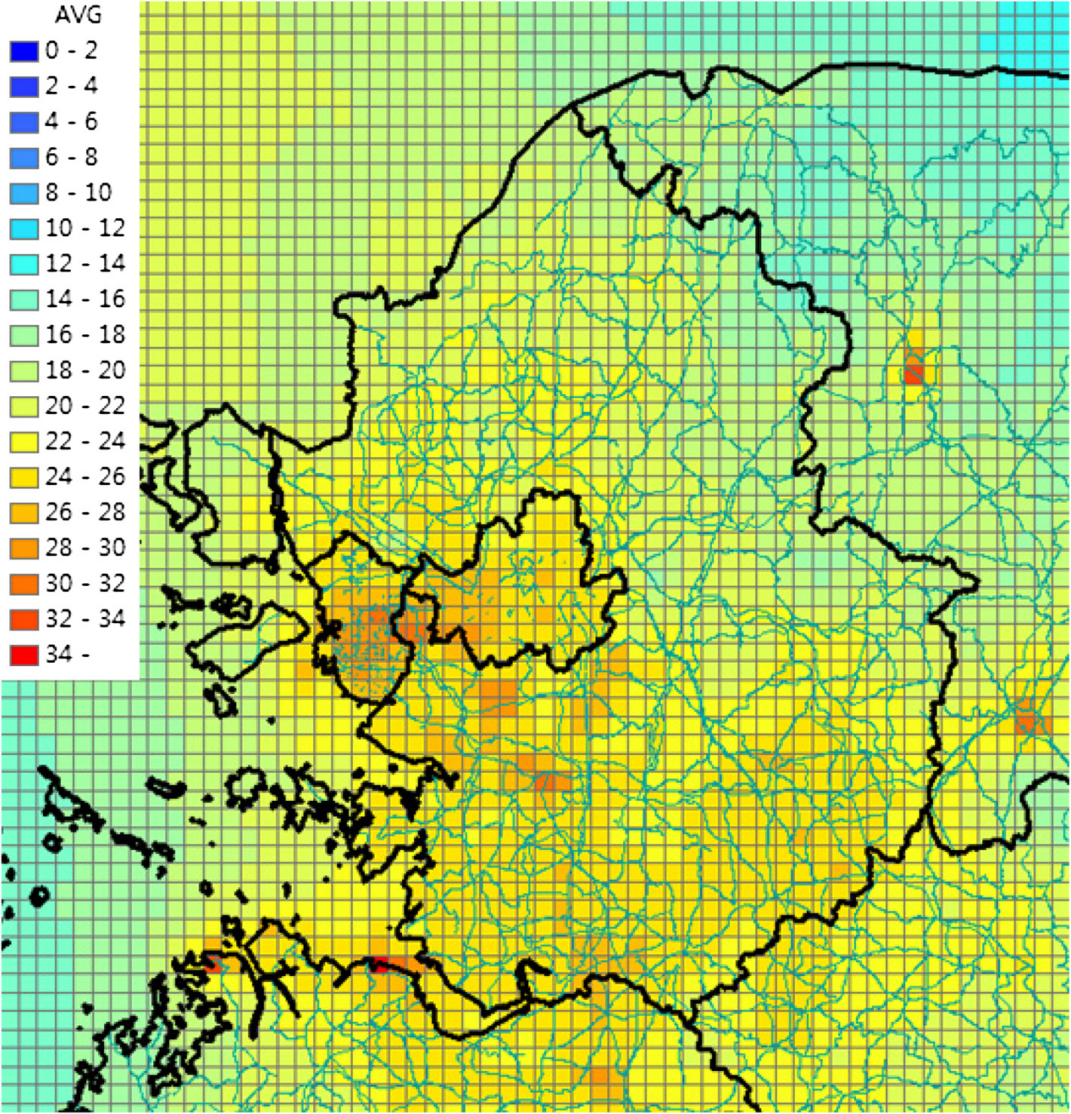
**PM2.5 concentration without reducing air emissions at 2024.**

**Figure 2:**
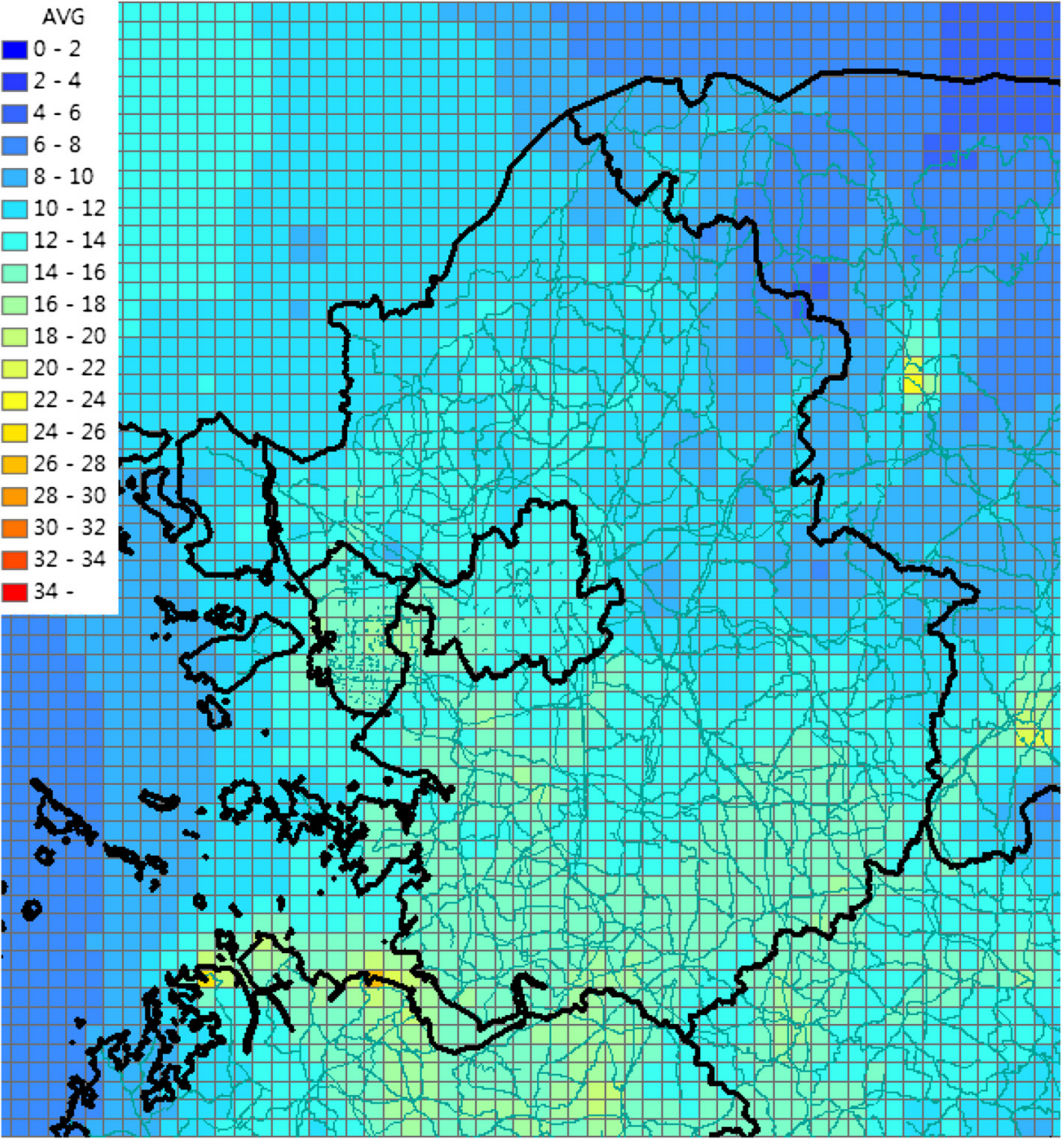
**PM2.5 concentration after reducing air emissions at 2024.**

**Figure 3:**
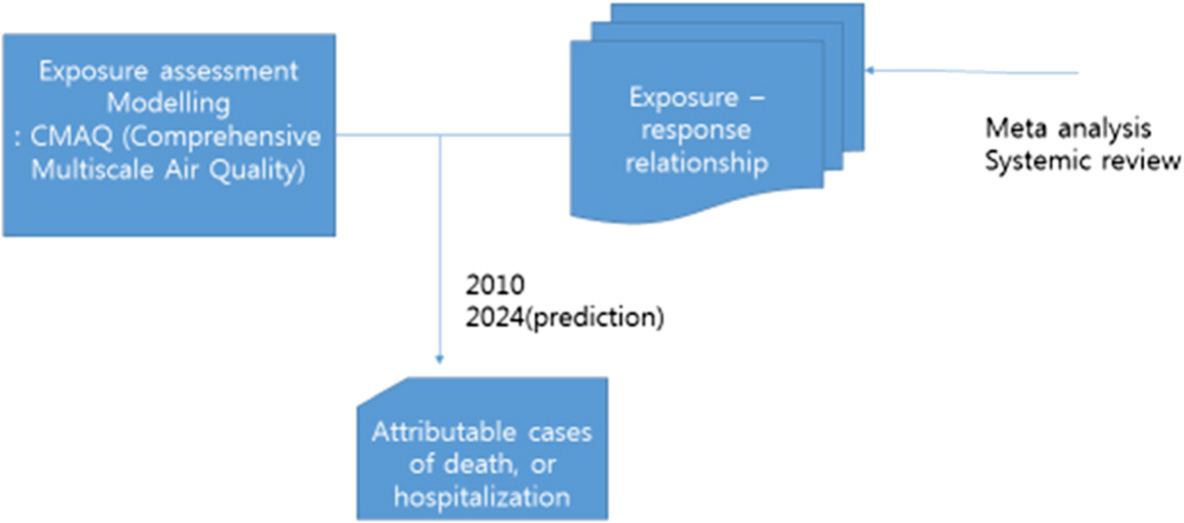
**Flow chart of this study.**

Table 2 summarizes the effect estimates, the specific health-outcome frequencies at E0, and the respective number of cases attributable to a 10 **μ**/m3 increase in PM_**10**_ (D10) for each health outcome. A summary of health outcomes attributed to particulate air pollution in the Seoul metropolitan area is shown in Table 3. The number of cases attributable to air pollution is given for three scenarios; year 2010, year 2024 without regulation regarding air pollution, year 2024, when the goal of air attainment is achieved. PM_**2.5**_ concentrations without reducing air emissions in 2024 are shown in Figure 1. PM_**2.5**_ concentrations after reducing air emissions in 2024 are shown in Figure 2. We compared the number of cases attributed to air pollution for three scenarios. We drew flow chart of this study (Figure 3).

**Table 3:** **Health outcome attributed to particulate air pollution in Seoul metropolitan area**

**Health outcome**	**2010**	**2024 Without regulation about air pollution**	**2024** **PM**_**2.5**_ **20 μg/m** ^**3**^ **PM**_**10**_ **30 μg/m** ^**3**^	**2024, the reducible number and fraction (%)**
	**95% CI**	**95% CI**	**95% CI**	
Premature deaths /persons/year)	15,346 (4,498 ~ 26,242)	25,781 (7,555 ~ 44,098)	10,866 (3,280 ~ 18,081)	14,915 (57.9%∇)
Respiratory hospital admissions (All age)	12,511 (956 ~ 23,583)	14,163 (1,114 ~ 26,701)	7,837 (611 ~ 14,865)	6,326 (44.7%∇)
Cardiovascular hospital admissions (All age)	12,351 (6,715 ~ 17,868)	13,982 (7,602 ~ 20,229)	7,736 (4,193 ~ 11,227)	6,246 (44.7%∇)
Lung cancer incidence	1,403 (254 ~ 2,170)	1,590 (287 ~ 2,462)	962 (159 ~ 1,613)	628 (45.2%∇)
Asthma attack (children < 18 years)	11,389 (7,180 ~ 15,601)	9,745 (6,143 ~ 13,350)	5,563 (3477 ~ 7,688)	4,182 (42.9%∇)
Asthma attack (adults ≥ 18 years)	44,006 (22,142 ~ 64,488)	53,300 (26,813 ~ 78,128)	29,789 (14,835 ~ 44,093)	23,511 (44.1%∇)
Chronic-bronchitis	20,490 (219 ~ 35,340)	24,836 (265 ~ 42,884)	14,276 (145 ~ 25,686)	10,560 (42.5%∇)
Acute bronchitis (age ≤ 18)	278,346 (153,949 ~ 360,290)	238,222 (131,744 ~ 313,483)	151,943 (78,491 ~ 212,851)	86,279 (36.2%∇)

Air pollution caused 15.9% of total mortality or approximately 15,346 attributable cases per year in 2010. Particulate air pollution also accounted for: 12,511 hospitalized cases of respiratory disease; 20,490 new cases of chronic bronchitis (adults); 278,346 episodes of acute bronchitis (children). After performing the 2^nd^ Seoul metropolitan air pollution management plan, Air pollution caused 6.7% of total mortality or approximately 10,866 attributable cases per year in 2024.

## Discussion

The public-health impact depends not only on the relative risk but also on the exposure distribution in the population. Our assessment assigned approximately 15.9% of annual deaths to outdoor air pollution. Our assessment is relatively high compared to 6% of EU’s assessment [12]. Korean people have high chronic disease incidences of cancer and chronic respiratory diseases, such as asthma and COPD [36]. According to OECD Health data [37], in 2009, the hospital admission rate for avoidable asthma in the population age 15 and over was 101.5/100,000 persons, while average OECD was 51.8/100,000 persons. In 2009, the hospital admission rate for COPD in the population age 15 and over was 222/100,000 persons, while average OECD was 198/100,000 persons. The incidence rate for all cancers combined in Korea showed an annual increase of 3.3% from 1999 to 2009 [38]. Considering current air pollution levels and our study’s results, certainly air pollution has important contribution to increases of cancer and chronic respiratory diseases in Seoul metropolitan area. Our study has strong advantages. First, our study attempted to decrease uncertainty inherent in these kinds of public health risk assessment. To account for inherent uncertainty in the impact assessment, an “at least” approach was applied for each step, and the uncertainties in the effect estimates were quantified and the results were given as a range (95% CI of the exposure-response function).

To assess the effects of air pollution—a complex mixture of pollutants—epidemiological studies use several indicators of exposure (e.g., NO_2_, CO, PM_10_, total suspended particles, SO_2_). However, because these pollutants are correlated, epidemiological studies cannot exactly allocate observed effects to each pollutant. A pollutant-by-pollutant assessment would grossly overestimate the impact. Therefore, we selected only one pollutant to derive the attributed cases.

The short-term effects of high pollution levels on mortality were not calculated separately because these are already included in exposure-response function of long-term mortality. We consider it inappropriate to use short-term studies for the impact assessment of annual mortality [39]. Short-term studies capture only part of air-pollution-related cases, namely those where exposure and event (death) are closely connected in time. Our calculation based on cohort studies captures both the short-term effects and the long-term effects. The number of deaths attributed to air pollution would be about 4–5 times smaller if the short-term effect estimates had been applied.

Second, we based our assumption on the consistency of epidemiological results observed across many countries. We derived exposure-response from selected well designed studies used in previous public health assessment. For mortality, we had to rely on two US studies, which were confirmed by a third US study [40], French PAARC study [41], China study [5]. Exposure-response used in our study would be consistent because it was confirmed across many countries.

Third, our study used nationwide frequency data. Health-outcome frequencies may strongly influence the impact assessment. Mortality from national sources may be considered accurate. However, frequency measures of morbidity and data on health-care systems have to be considered estimates with some inherent uncertainties. We selected national health frequency data in order to reduce the impact of these limitations. National health insurance covers almost all people in Korea, so that health-outcome frequencies in our study have fewer inherent uncertainties.

However, our study has some limitations. First, our assessment relies on some limited study for deriving exposure-response functions. For chronic bronchitis, our assessment relies on one study. The advantage of the study is the reporting of effects of PM on the incidence of chronic bronchitis among a population with very low rates of smoking. This measure was particularly useful for the economic valuation and had been used in other studies [42]. Second, our study restricted the effect of air pollution to PM_10 and_ PM_2.5_, and did not take other pollutants, such as NOx, SO_2_ and O_3_ into consideration. It can underestimate independent effects of air pollution not explained by or correlated by PM fractions. Temperature is increasing due to climate change. Ozone, showing higher concentration these days, is expected to have more hazardous effects on mortality in Korea and other countries [43] than before. Our calculation regarding the effect of air pollution may be underestimated, because we consider the effect of PM only.

As we did not quantify the attributed number of deaths below age 30 years, we might have underestimated the real number of deaths attributed to air pollution. We ignored potential effects on newborn babies or infants [44]. Although infant mortality is low in Korea, and thus the number of attributed cases is small, the impact on years of life lost, and therefore the economic valuation, could be considerable.

Third, we did not consider uncertainty in exposure assessment. If we assume another exposure reference value, the impact estimates will be higher. Our study assumed that the health risk showed the least at PM_10_ exposure level <20 μg/m^3^. In Korea, we have never experienced PM_10_ exposure level <20 μg/m3. In other countries such as EU and US, an exposure level of 15 μg/m^3^ corresponds to the reference value in the public-health impact [45]. Our study can be underestimated because of our assumption about the reference value.

Apart from the variability of epidemiological exposure-response estimates (95% CI), we did not quantify other sources of uncertainty, such as errors in the population exposure distribution, or in the estimation of health outcome frequencies. Simulations of multiple probability distributions may, however, erroneously suggest a level of precision in assessing uncertainty that cannot be achieved. These kinds of public health assessment contained a lot of uncertainty. Our study took an “at least” approach in order to account for inherent uncertainty in the impact assessment. Our study insisted that the risk attributed to air pollution in Korea at least exceeded our assessment.

Recent publication about air pollution study in Korea focused on short-term effects of air pollution [46,47]. Recently some studies report adverse pregnancy outcomes associated with air pollution from birth cohort study [48,49]. There is no report like this study in Korea. We assessed public health benefit by calculating mortality and morbidity cases attributed to air pollution in the Seoul metropolitan area.

Our study showed that air pollution had the significant impact on the health of Korean people. In particular, elderly people and children are the vulnerable population to air pollution. In the valuation of air pollution related death and hospitalization, assumptions about age structure of those affected may be influential [47]. The affected population will increase because of the rapidly ageing population structure. Korea is a rapidly aging society, and the number of elderly people older than 65 years is rapidly increasing. Actually the number of elderly people older than 65 years in 2024 will be 12,635,000 persons and their portion will be 24.4% among all population. Traffic is important contributor to urban air pollution in Korea and Asian countries. But traffic creates costs that are not covered by the polluters. The real related external costs from the Organization for Economic Cooperation and Development (OECD) are quantified [50-53]. The traffic share of the total PM_10_ exposure depended on the mean concentration, ranging from 28% at an annual mean PM_10_ of 10–15 μg/m^3^, and increasing up to 58% in some areas. In Korea, the traffic share of the total PM_2.5_ exposure will be higher due to rapid urbanization. Traffic air pollution will be great burden to urban air pollution because if increasing car numbers in the near future.

Death attributed to air pollution will increase, if proper countermeasures are not taken. If PM_2,5_ can be maintained at less than 20 ug/m^3^ in 2024 in the Seoul metropolitan area, death attributable to air pollution will decrease from 25,781 to 10,866. Korean people have a high disease burden of cancer and chronic respiratory diseases, such as asthma and COPD, attributed to air pollution. Clean air strategies, such as the 2^nd^ air management plan in the Seoul metropolitan area, will decrease the burden of disease in Korean people.

Even after accounting for the overall uncertainty of this estimation, our study emphasizes the need to consider air pollution as a pivotal cause of impaired health. In a century moving toward a sustainable society, closer collaboration of public health and environmental policies will enable us to have preventive capacity. Further development of standardized impact assessment methods is needed in order to more stringently assess the benefits from clean air strategies.
